# Lack of association between serum syndecan-4, myocardial fibrosis and ventricular dysfunction in subjects with chronic Chagas disease

**DOI:** 10.1371/journal.pone.0189408

**Published:** 2017-12-12

**Authors:** Ticiana Ferreira Larocca, Carolina Thé Macêdo, Márcia Noya-Rabelo, Luís Cláudio Lemos Correia, Moisés Imbassahy Moreira, Alessandra Carvalho Caldas, Jorge Andion Torreão, Bruno Solano de Freitas Souza, Juliana Fraga Vasconcelos, Alexandre Schaer Carvalho da Silva, Ricardo Ribeiro dos Santos, Milena Botelho Pereira Soares

**Affiliations:** 1 Center for Biotechnology and Cell Therapy, São Rafael Hospital, Salvador, Bahia, Brazil; 2 Gonçalo Moniz Institute, IGM-Fiocruz/BA, Salvador, Bahia, Brazil; 3 Department of Cardiology, São Rafael Hospital, Salvador, Bahia, Brazil; 4 Escola Bahiana de Medicina e Saúde Pública, Salvador, Bahia, Brazil; University of Sao Paulo, BRAZIL

## Abstract

**Background:**

Syndecan-4 is a transmembrane glycoprotein associated with inflammation and fibrosis. Increased syndecan-4 levels were previously detected after acute myocardial infarction and in subjects with heart failure. However, the levels of syndecan-4 in subjects with Chagas disease have not so far been investigated. The aim of this study was to investigate the potential role of serum sydencan-4 as a novel biomarker for myocardial fibrosis and cardiac dysfunction in subjects with Chagas disease.

**Methods:**

This study comprised subjects with Chagas disease (n = 56), being 14 (25%) with the indeterminate form, 16 (29%) with the cardiac form without ventricular dysfunction, and 26 (46%) with the cardiac form with ventricular dysfunction.

**Results:**

Syndecan-4 serum concentrations did not correlate with presence or absence of myocardial fibrosis (P = 0.386) nor disease severity in subjects with Chagas disease (P = 0.918). Additionally, no correlation was found either between the degree of myocardial fibrosis and serum syndecan-4 [r = 0.08; P = 0.567] or between left ventricular ejection fraction and syndecan-4 [r = 0.02; P = 0.864]. In contrast, NT-proBNP levels correlated with ejection fraction and myocardial fibrosis.

**Conclusions:**

Our results demonstrate the lack of correlations between serum syndecan-4, myocardial fibrosis and cardiac dysfunction in subjects with Chagas disease. Further studies are required to show if syndecan-4 concentrations can be marker for prognosis assessment or disease progression.

## Introduction

Chagas disease (CD), caused by infection with the protozoan parasite *Trypanosoma cruzi*, is a major public health problem in Latin America [1] and is becoming an emerging problem in non-endemic areas due to population migration [2,3]. Subjects with Chagas disease can be classified in two large groups: those with the indeterminate form, without clinical symptoms or abnormalities in routine tests, and those with the determinate form, with symptoms, abnormal tests, or both [4]. Most of the cases in the chronic phase present as the indeterminate form, but after years or decades they may evolve to the determinate form, presenting either cardiac, digestive, or cardio-digestive symptoms [5]. Chronic Chagas disease cardiomyopathy (CCC), the most severe form of clinical presentation in CD, has worse long-term prognosis when compared to cardiomyopathies due to other etiologies [6].

The hallmark of CCC is the presence of a multifocal inflammatory reaction leading to myocardial fibrosis, often followed by ventricular dysfunction and arrhythmias [5,7,8]. At this disease stage, there is no effective treatment other than heart transplantation. Since the cardiac form of Chagas disease occurs in approximately 30% of the infected patients, and usually develops during 10–30 years after the acute phase, it is of great interest to identify biomarkers that might be used for early detection of cardiac involvement [6].

Previously, we performed a transcriptomic analysis and demonstrated that several genes associated with inflammation and fibrosis, including syndecan-4, are overexpressed in the hearts of mice chronically infected with *Trypanosoma cruzi* [9]. Syndecan-4 is the most studied member of the syndecan family, composed by transmembrane proteins capable of carrying heparan sulfate and chondroitin sulfate chains. Its activities include modulation of fibroblast growth factor signaling, regulation of cell migration and control of cell adhesion. Although syndecan-4 is expressed by several cell types [10], it is mainly expressed in endothelial cells, smooth muscle cells and cardiomyocytes [11]. It is already known that the expression of the syndecans can be altered under certain pathophysiological conditions, including mechanical stress [12] and tumor metastasis [13], in response to growth factors present in the tissue microenvironment, such as FGF2, TNF-α and TGF-β [14–16].

Recently, evidence pointing to the involvement of syndecan-4 in the pathogenesis of cardiovascular diseases has emerged. Circulating syndecan-4 concentrations are increased after acute myocardial infarction [17] and also in heart failure, inversely correlated with left ventricular ejection fraction [18], suggesting a possible role as a predictive biomarker of cardiovascular events [19]. However, serum syndecan-4 concentrations have neither been evaluated in CD subjects nor correlated with significant predictors of outcomes in this group of patients. Thus, the aim of this study was to evaluate the potential role of serum syndecan-4 as a novel biomarker for disease severity in CD, by concentrations with myocardial fibrosis and left ventricular ejection fraction (LVEF). Additionally, syndecan-4 serum concentrations were compared between subjects presenting different clinical forms of CD.

## Materials and methods

### Study population

We conducted an observational study in which subjects with CD were enrolled. Between January 2011 and December 2013, we enrolled 56 subjects in the CD outpatient clinics at our institution. All subjects with Chagas disease were classified in groups according to the clinical form of presentation, as follows: indeterminate form (subjects with no evidence of cardiac involvement or heart failure), cardiac form without ventricular dysfunction and cardiac form with ventricular dysfunction.

Inclusion criteria for CD subjects were based on confirmation by two positive serologic tests (indirect hemagglutination and indirect immunofluorescence), and age (18–70 years). Exclusion criteria for subjects with CD were previous myocardial infarction or history of coronary artery disease, primary valve disease, terminal renal failure dialysis treatment, active liver disease, hematologic, neoplastic or bone diseases and magnetic resonance imaging contraindications.

We obtained structured medical history, and all CD subjects underwent physical examination, blood analysis, 12-lead electrocardiogram, chest X-Ray, 24-h Holter monitoring, conventional Doppler echocardiogram, and cardiovascular magnetic resonance imaging. All patients with heart failure and/or arrhythmias received the standard therapy with diuretics, beta-blockers, ACE inhibitors, angiotensin II receptor blockers, digoxin, or amiodarone, as appropriate.

Study complied with the Declaration of Helsinki, was approved by the Ethics Committee of the São Rafael Hospital, and is registered in ClinicalTrials.gov under the identifier NCT01842867. All subjects signed written informed consent before study inclusion.

### Measurement of syndecan-4 and NT-ProBNP

Serum syndecan-4 concentration was measured by a solid phase sandwich ELISA, according to the manufacturer’s instructions (IBL Co., Ltd., Fujioka, Japan). This ELISA system recognizes the secreted ectodomain of the syndecan-4 molecule in the blood by using two highly specific antibodies. Tetramethylbenzidine (TMB) was used as chromogen and the strength of coloring was proportional to human syndecan-4 amount. The sensitivity of the test was 3.94 pg/mL. Plates were read at 450 nm using a multiplate reader (EnVision, PerkinElmer).

Plasma NT-ProBNP measurements were performed with an automated quantitative enzyme linked fluorescent assay (VIDAS^®^ NT-proBNP2; bioMérieux, France), according to the manufacturer’s instructions.

### Doppler echocardiogram

Standard transthoracic echocardiographic examinations were recorded in all subjects using the Vivid 7 digital ultrasound system (GE Vingmed Ultrasound AS, Horten, Norway). Three cardiac cycles were stored in cine loop format for online analysis. Left ventricle and left atrial dimensions were measured according to the American Society of Echocardiography’s recommendations [20]. The LVEF was measured using the biplane Simpson’s method. Diastolic function was evaluated by the analysis of the mitral Doppler inflow, pulsed-wave DTI at the lateral mitral annulus, and mitral propagation velocity using color M-mode echocardiography. Mitral regurgitation was obtained using the proximal isovelocity surface area method. Right ventricular function was assessed with the S0 systolic velocity in the lateral wall.

### Magnetic resonance imaging

Cardiovascular magnetic resonance imaging (MRI) was performed using a Sigma HDx 1.5-T system (General Electric; Fairfield, CT, USA). For assessment of the LV function, electrocardiography-gated, breath-hold long-axis, short-axis, and four-chamber views were acquired in the same location in different sequences. Acquisition parameters used for the dynamic sequence included a repetition time (RT) of 3.5 ms, an echo time (ET) of 1.5 ms, flip angle of 60°, a receiver bandwidth of 125 kHz, a 35 x 35 cm field of view (FOV), a matrix of 256x148, a temporal resolution (TR) of 35 ms and a slice thickness of 8.0 mm without gap.

Delayed enhancement (DE) images were acquired every heartbeat, 10 to 20 min after the administration of a gadolinium-based contrast (0.1 mmol/kg) using RT of 7.1 ms, ET of 3.1 ms, flip angle of 20°, first cardiac phase, views per segment 16/32, matrix size of 256 x 192, slice thickness of 8.0 mm, gap between slices of 2 mm, field of view 32 to 38 cm, inversion time 150 to 300 ms, receiver bandwidth of 31.25 kHz, number of excitations of 2. The myocardial DE technique was used to investigate myocardial fibrosis, which was estimated by a quantitative visual method [7].

### Statistical analysis

Categorical data were presented as numbers (percentages), and continuous data were expressed as mean ± SD or median (interquartile range). Comparisons of continuous variables among groups were performed with analysis of variance (ANOVA) test or Kruskal-Wallis, depending on normality assessed by Shapiro-Wilk test. Chi-Square or Fisher tests were applied for proportions comparisons. Correlations between continuous variables were evaluated by Pearson or Spearman coefficients, depending on normality. Cases with missing data were not included in the analysis. Analyses were performed using SPSS version 20.0 (IBM), and p < 0.05 (two-tailed) was considered statistically significant.

## Results

### Baseline characteristics

The study included 56 subjects with Chagas disease, 41% were men, and the mean age was 58±9 years. Regarding the clinical form of presentation, the subjects were distributed as follows: 14 (25%) with the indeterminate form (subjects with no evidence of cardiac involvement or heart failure), 16 (29%) with the cardiac form without ventricular dysfunction and 26 (46%) with the cardiac form with ventricular dysfunction. The median for NT-ProBNP level was, respectively, 60.5 pg/ml (IIQ: 32.8–109.8), 92.50 pg/ml (IIQ: 62.3–180.5) and 816 pg/ml (IIQ:196.5–2128) p < 0.001. Other clinical and demographic characteristics of the subjects with Chagas disease are described in Table 1.

**Table 1:** Clinical and demographic characteristics of subjects with Chagas disease.

Form	Indeterminate	Cardiac without dysfunction	Cardiac with dysfunction	p
n	14	16	26	
Male gender	5 (36)	4 (25)	14 (54)	0.181
Age (years)	60 ± 10	56 ± 8	59 ± 9	0.670
Black or mullato self-reported race	13 (93)	14 (88)	23 (88)	1.00
NYHA III or IV	0	0	14 (54)	<0.001
Hypertension	11 (79)	12 (75)	16 (62)	0.332
Diabetes	3 (21)	0	5 (19)	0.253
Current smoking	2 (14)	3 (19)	9 (35)	0.371
Hypercholesterolemia	6 (43)	8 (50)	11 (42)	0.645
RBBB	0	15 (94)	14 (54)	<0.001
Cardiomegaly (x-Ray)	2* (14)	6 (38)	19 (73)	0.001
VT	0	3 (19)	14 (54)	0.002
NT-ProBNP (pg/ml)	60.5 (32.8–109.8)	92.5 (66.3–180.5)	839.5 (239.8–2271)	<0.001
hsPCR	1.1 (0.2–3.8)	1.2 (0.5–4.7)	1.1 (0.7–3.3)	0.75
Hemoglobin (g/dl)	14.1 ± 1.1	13.7 ± 0.9	14.2 ± 0.9	0.167
Creatinin (mg/dl)	0.83 ± 0.16	0.81 ± 0.12	0.93 ± 0.18	0.030

Data are expressed as mean ± SD or median (interquartile interval) for continuous variables, or number (%) for discrete variables. hsCRP = highly sensitive C Reactive Protein. NYHA = New York Heart Association; RBBB = right bundle-branch block; VT = ventricular tachycardia.

*Cardiomegaly not confirmed by echocardiography.

### Cardiovascular imaging characteristics

The mean LVEF assessed by Simpson was 53 ± 15% for all subjects with Chagas disease, and 39 ± 12% for the subjects with the cardiac form with ventricular dysfunction.

Delayed enhancement was identified in 35 subjects (66%), and the total amount of myocardial fibrosis was 9% (IIQ: 2.4–18). Myocardial fibrosis was detected in 6 of 14 (42%) subjects in the indeterminate form (median 4.1%, IIQ: 2.1–10.8), 7 of 16 (43%) in the cardiac form without ventricular dysfunction (median 2.3%, IIQ: 1.0–5.0) and 22 of 26 (85%) subjects in the cardiac form with ventricular dysfunction (median 15%, IIQ: 7.3–25.6, P = 0.001). Echocardiographic and MRI data for subjects with Chagas disease are described in Table 2. Echocardiographic data for all subjects with heart failure are shown in Table 3.

**Table 2:** Echocardiographic and CMR findings in subjects with Chagas disease.

Form	Indeterminate	Cardiac without dysfunction	Cardiac with dysfunction	p
Echocardiogram*				
LVEF (%)	64.5 ± 3.5	64.7 ± 3.9	39 ± 12.3	<0.001
LV ESV (ml)	33 ± 7.1	36 ± 12.3	136 ± 67	<0.001
LV EDV (ml)	129.8 ± 22.6	115.8 ± 22	240.3 ± 83.5	<0.001
LV mass (g)	136.8 ± 28.5	134.6 ± 34.8	215.3 ± 58.7	<0.001
Diastolic dysfunction				
Type I	6 (43)	6 (38)	14 (58)	0.038
Type II	0	1 (7)	6 (25)	0.007
Type III	0	0	0	
Tei index	0.53 ± 0.08	0.50 ± 0.1	0.61 ± 0.19	0.341
E/E′ ratio	8.5 ± 1.8	8.1 ± 3.0	12.9 ± 8.0	0.018
Tricuspid annular systolic velocity (cm/s)	13 ± 2.3	12 ± 1.7	12.1 ± 1.8	0.032
MRI**				
LVEF (%)	70.9 ± 8.4	67.0 ± 6.6	35.3 ± 15.3	<0.001
RVEF (%)	65.2 ± 9.5	58.8 ± 9.6	46.8 ± 14.9	<0.001
LV ESV (ml/m^2^)	35.2 ± 13.3	40.5 ± 15.3	148.9 ± 86.2	<0.001
LV EDV (ml/m^2^)	118.8 ± 22.6	119.9 ± 29.8	222.9 ± 79.2	<0.001
LV mass (g/m^2^)	111.6 ± 30.2	93.6 ± 29.8	150 ± 60.4	0.004
Fibrosis (%)‡	4.1 (2.1–10.8)	2.3 (1.0–5.0)	15.0 (7.3–25.6)	0.001
Delayed enhancement	6 (42)	7 (43)	22 (85)	0.006

Data are expressed as mean ± SD or median (interquartile interval) or number (%) for discrete variables. EDV = end diastolic volume; ESV = end systolic volume; LV = left ventricular; LVEF = left ventricular ejection fraction; MRI = magnetic resonance imaging; RVEF = right ventricular ejection fraction.

* Data from 55 subjects.

** Data from 53 subjects.

^‡^ Data from subjects with delayed enhancement on MRI (n = 35).

**Table 3:** Echocardiographic data in subjects with heart failure.

n	26
LVEF (%)	39 ± 12.3
LV mass (g)	215.3 ± 58.7
Left atrium (mm)	40 ± 5.9
Diastolic dysfunction	
Type I	14 (58)
Type II	6 (25)
Type III	0

Data are expressed as mean ± SD or number (%) for discrete variables. LV = left ventricular; LVEF = left ventricular ejection fraction.

### Assessment of syndecan-4

**Fig 1:**
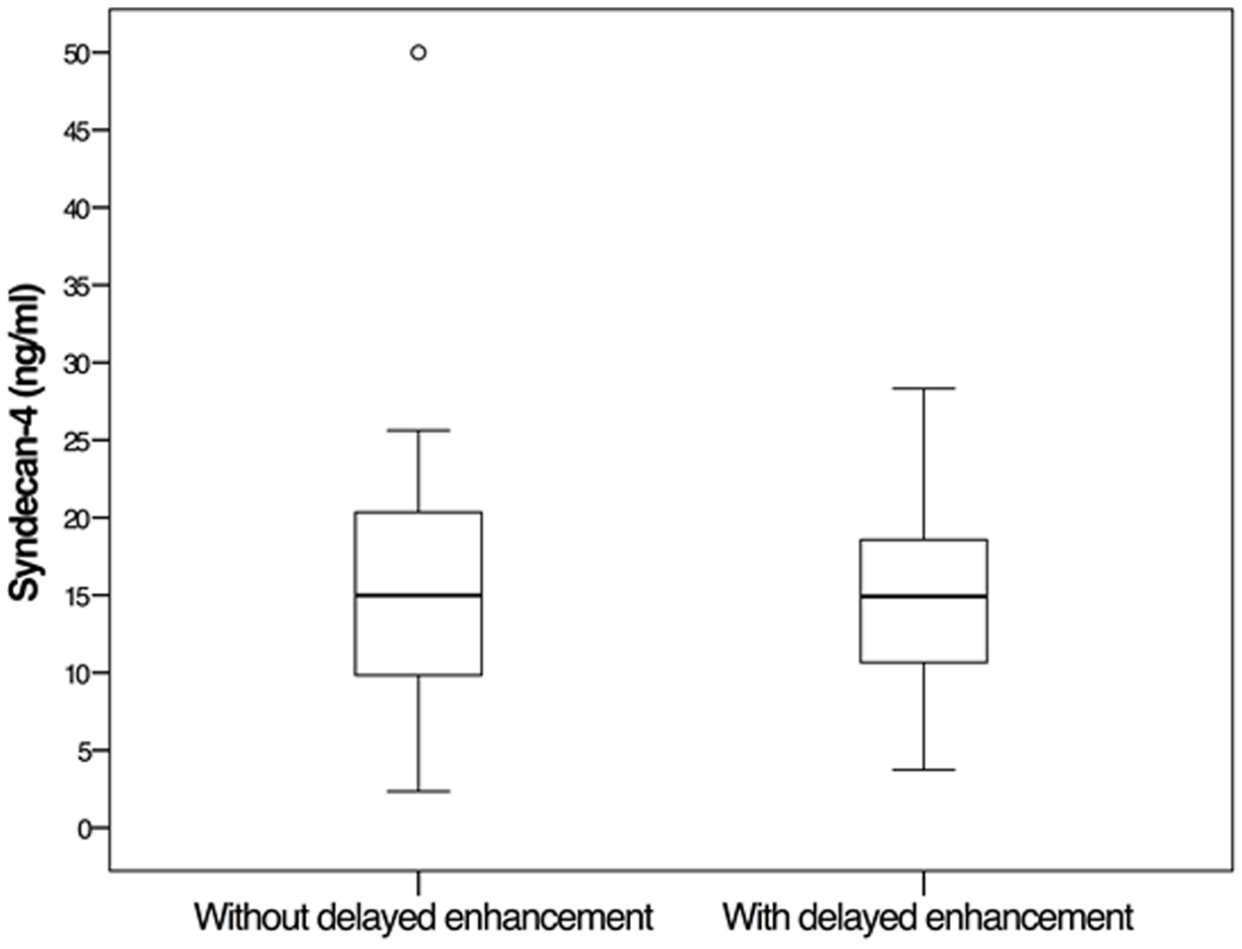
Serum concentration of syndecan-4 assessed by ELISA in subjects with and without delayed enhancement detected by magnetic resonance imaging. Circle = outlier, * = extreme outlier, *t* test, P = 0.386.

The mean concentration of syndecan-4 in subjects with Chagas disease was 15.4 ± 7.7 ng/ml. No correlation was observed between syndecan-4 concentrations and myocardial fibrosis, with a mean syndecan-4 concentration of 14.9 ± 5.8 ng/ml in subjects with DE versus 16.8 ± 10.2 ng/ml in subjects without DE (P = 0.386; Fig 1).

**Fig 2:**
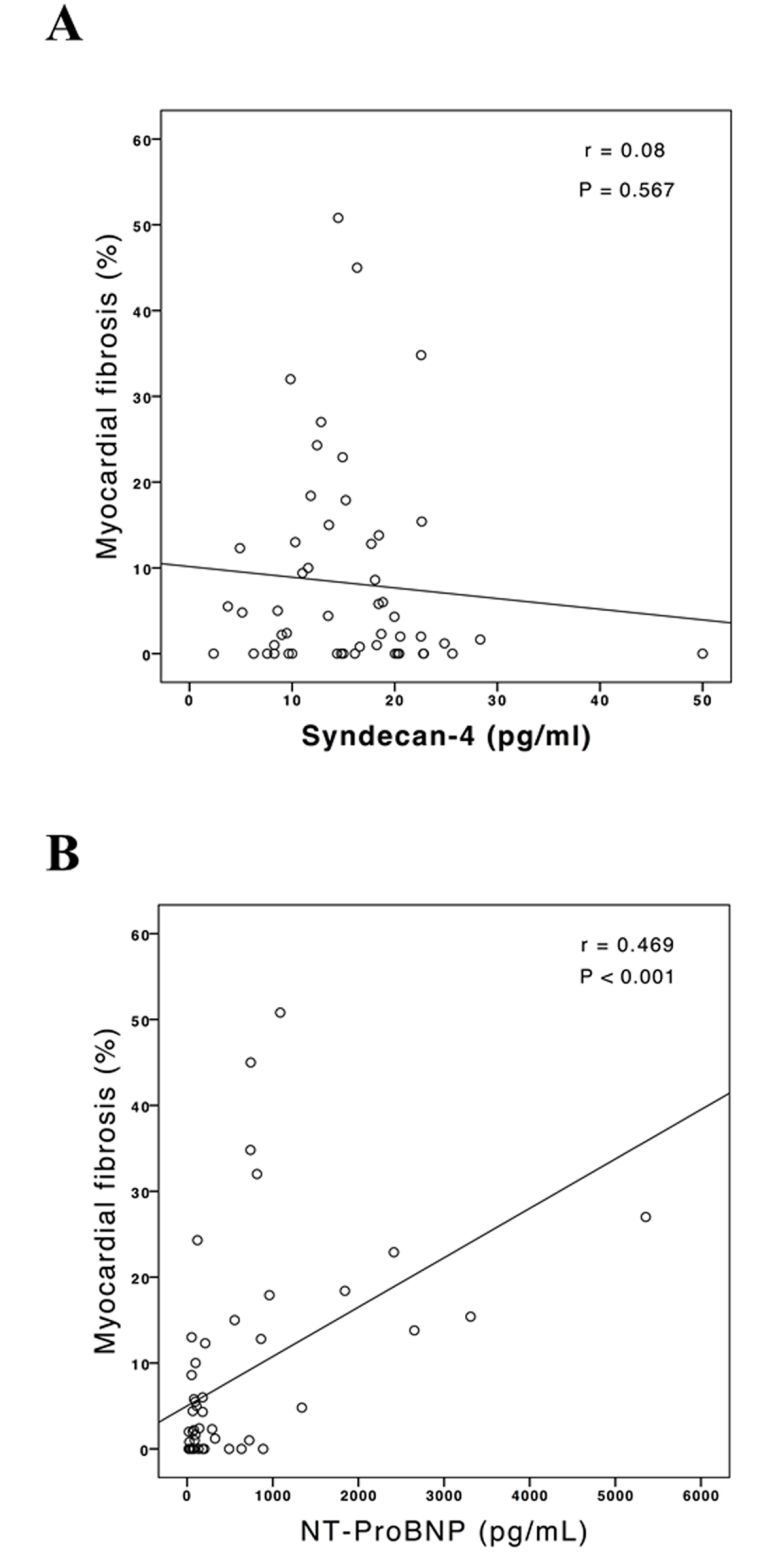
Correlation between percentage of myocardial fibrosis assessed by cardiovascular MRI and concentration of syndecan-4 and NT-ProBNP. A: Pearson’s correlation, r = 0.08, P = 0.567. B: Pearson’s correlation, r = 0.469, P<0.001.

**Fig 3:**
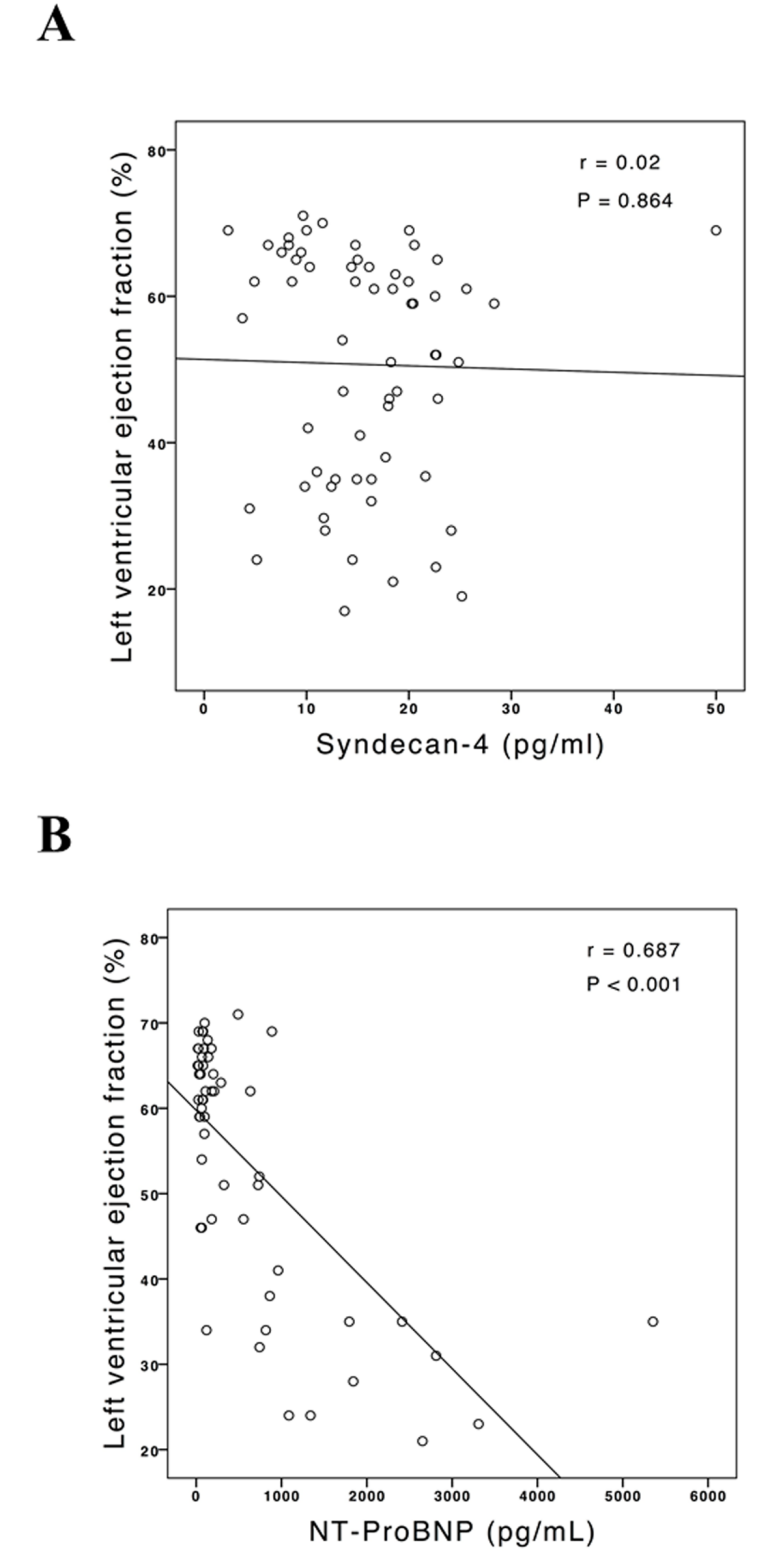
Correlation between LVEF and serum concentration of syndecan-4 and NT-ProBNP. A: Pearson’s correlation, LVEF assessed by Simpson’s method, r = 0.02, P = 0.864. B = Pearson’s correlation, LVEF assessed by Simpson’s method, r = 0.687, P<0.001.

No correlation was found either between myocardial fibrosis and syndecan-4 (r = 0.08, P = 0.567; Fig 2A) or between LVEF and syndecan-4 (r = 0.02, P = 0.864; Fig 3A). As expected, a moderate to strong correlation was found between myocardial fibrosis and NT-ProBNP (r = 0.47, p < 0.001; Fig 2B), and also between LVEF and NT-ProBNP, as a negative correlation (r = 0.69, p < 0.001; Fig 3B).

**Fig 4:**
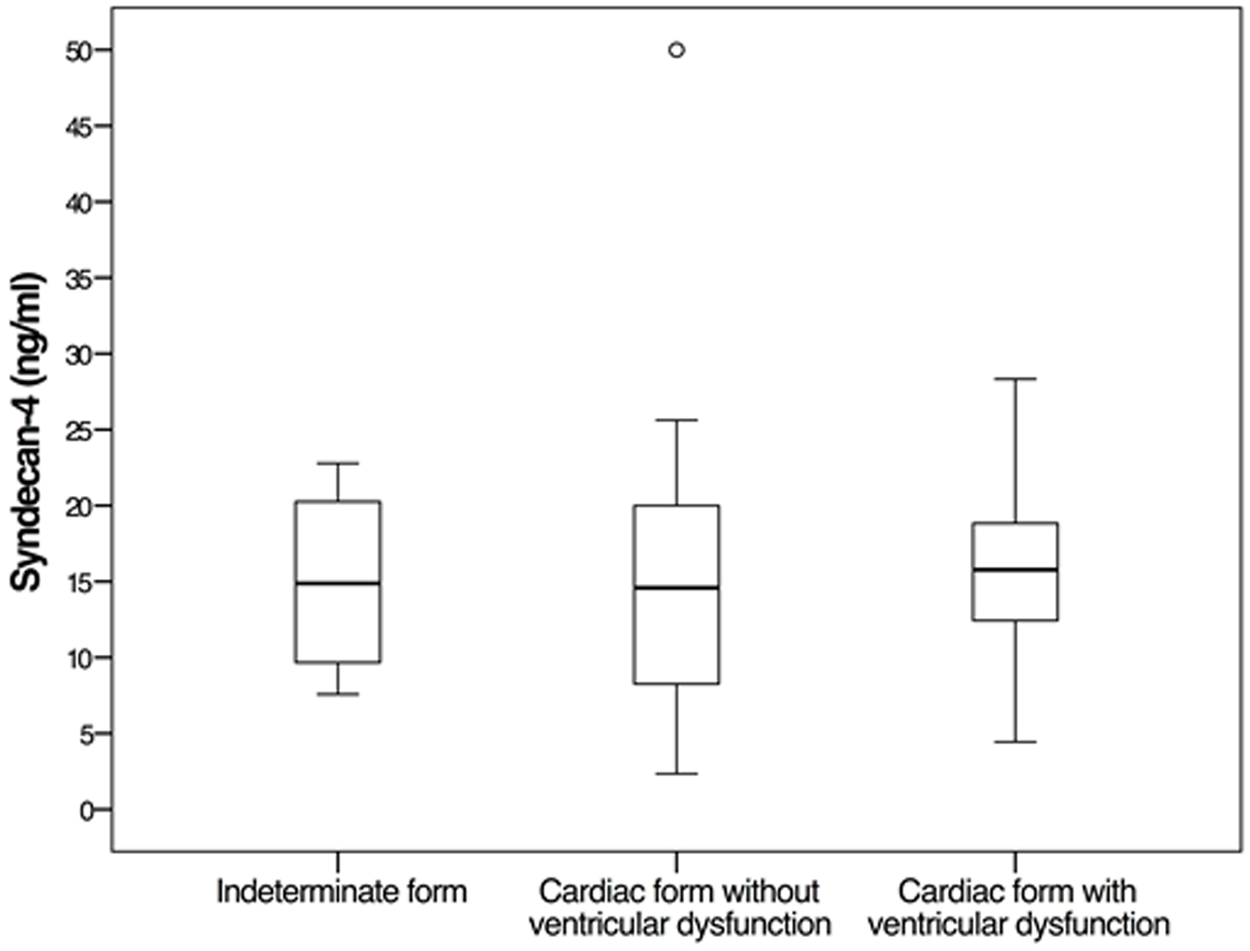
Serum concentration of syndecan-4 assessed by ELISA in subjects with different forms of Chagas disease. ANOVA, P = 0.918. Circle = outlier, * = extreme outlier, P = 0.395.

No differences in syndecan-4 concentrations were detected comparing groups of subjects with different forms of Chagas disease. In subjects with the indeterminate form, the mean syndecan-4 concentration was 14.7 ± 5.1 ng/ml; in subjects with cardiac form without ventricular dysfunction, 15.6 ± 11.5 ng/ml; and in subjects with cardiac form with ventricular dysfunction, 15.7 ± 6.0 ng/ml (P = 0.918; Fig 4).

## Discussion

According to Morrow and de Lemos, a good biomarker for clinical use must fulfill three main criteria. First, the assay for its measurement should be easily accessible for a reasonable cost. Second, it must add information to already existing tests. And third, there must be strong evidence that it is important to managing disease progression or response to therapy [21]. In the current study, we provided the first evaluation of syndecan-4 as a biomarker for heart disease severity in a population with Chagas disease.

Previously, we showed that syndecan-4 is overexpressed in hearts of mice chronically infected with *Trypanosoma cruzi* in experimental model of Chagas disease cardiomyopathy [9]. In the present study, however, no increase in serum concentration of syndecan-4 was observed in subjects with chronic Chagas disease cardiomyopathy, regardless of disease severity. This could imply that syndecan-4 is not involved in the pathogenesis of the disease in humans. Therefore, additional studies in human heart samples may help clarify whether syndecan-4 expression is increased in the heart tissue in individuals with chronic Chagas cardiomyopathy, as seen in the experimental model of Chagas disease.

It was previously reported that, in subjects with dilated cardiomyopathy due to other etiologies, serum levels of syndecan-4 correlated negatively with LVEF and diastolic diameters [22]. However, we did not find any correlation between syndecan-4 and left ventricular systolic or diastolic diameters in our study. Additionally, Takahashi and colleagues assessed the concentration of syndecan-4 in 45 subjects with heart failure and 21 healthy subjects, finding also a moderate and negative correlation between syndecan-4 and LVEF [19], suggesting that, like NT-ProBNP, it might be a useful biomarker for heart failure [23].

Despite the lack of correlation between syndecan-4 and cardiac disease severity, our analysis in the clinical setting of Chagas disease showed a moderate to strong correlation between LVEF and concentration of NT-ProBNP. It is noteworthy that, in the present study, we used the same assay employed in a previous study for measuring syndecan-4 [19], that reported association between syndecan-4 levels and cardiac disease severity.

Our study showed a high percentage of delayed enhancement in the three different forms of the disease: 42% for the indeterminate form, 43% for the cardiac form without ventricular dysfunction and 85% for subjects with ventricular dysfunction. A previous study demonstrated rates of 7.4%, 15.8% and 52.4% for these three groups of subjects, respectively [24]. Despite this difference, the overall prevalence of delayed enhancement in our population of subjects with chronic Chagas cardiomyopathy is consistent with literature data, since global prevalence has demonstrated delayed enhancement ranging from 68.6% to 88.9% in this profile of subjects [7].

Since it was already demonstrated the relationship between syndecan-4 and granulation tissue formation and wound repair in experimental model of myocardial infarction [25], we expected to find a high concentration of syndecan-4 in subjects with cardiac forms of Chagas disease, for which the hallmark is the fibrosis formation. The concentration of syndecan-4, however, was not different among subjects with different forms of Chagas disease. Additionally, no correlation was found between the percentage of myocardial fibrosis and syndecan-4 concentration.

Ethnicity is a known factor that influences the predictive values of biomarkers in the cardiovascular field [26]. Indeed, the studies evaluating syndecan-4 as a heart failure biomarker published so far include mostly Asians or Caucasian populations, and data from mixed, and afro-descendent populations, such as the one included in our study, cannot be found in the literature. Similarly, galectin-3, another biomarker that has been studied in the field of heart failure, was shown to have a prognostic value for heart in Caucasians, but not in an afro-descendent population [27]. Plasma levels of galectin-3 also did not correlate with disease severity in Chagas disease patients [28].

In conclusion, our study showed a lack of correlation between either the degree of myocardial fibrosis or the LVEF and the serum concentration of syndecan-4 in subjects with Chagas disease. The current study has limitations, including being conducted in a single tertiary and academic hospital, with a small sample size, and being a cross-sectional study with a single measurement of syndecan-4. Studies with larger number of patients are required to determine the influence of ethnicity on the predictive role of syndecan-4 as a biomarker for heart failure of different etiologies. All supporting information for this study can be found in S1 Table.

## Supporting information

S1 Table Dataset for the syndecan-4 study. (XLS)
